# A clinical guide to hereditary cancer panel testing: evaluation of gene-specific cancer associations and sensitivity of genetic testing criteria in a cohort of 165,000 high-risk patients

**DOI:** 10.1038/s41436-019-0633-8

**Published:** 2019-08-13

**Authors:** Holly LaDuca, Eric C. Polley, Amal Yussuf, Lily Hoang, Stephanie Gutierrez, Steven N. Hart, Siddhartha Yadav, Chunling Hu, Jie Na, David E. Goldgar, Kelly Fulk, Laura Panos Smith, Carolyn Horton, Jessica Profato, Tina Pesaran, Chia-Ling Gau, Melissa Pronold, Brigette Tippin Davis, Elizabeth C. Chao, Fergus J. Couch, Jill S. Dolinsky

**Affiliations:** 1 Ambry Genetics, Aliso Viejo, CA USA; 2 Department of Health Sciences Research, Mayo Clinic, Rochester, MN USA; 3 Department of Medical Oncology, Mayo Clinic, Rochester, MN USA; 4 Department of Laboratory Medicine and Pathology, Mayo Clinic, Rochester, MN USA; 5 Department of Dermatology, University of Utah, Salt Lake City, UT USA; 6 Department of Pediatrics, Division of Genetic and Genomic Medicine, University of California–Irvine, Irvine, CA USA

**Keywords:** hereditary cancer, cancer predisposition, multigene panel, testing criteria, clinical validity

## Abstract

**Purpose:**

Despite the rapid uptake of multigene panel testing (MGPT) for hereditary cancer predisposition, there is limited guidance surrounding indications for testing and genes to include.

**Methods:**

To inform the clinical approach to hereditary cancer MGPT, we comprehensively evaluated 32 cancer predisposition genes by assessing phenotype-specific pathogenic variant (PV) frequencies, cancer risk associations, and performance of genetic testing criteria in a cohort of 165,000 patients referred for MGPT.

**Results:**

We identified extensive genetic heterogeneity surrounding predisposition to cancer types commonly referred for germline testing (breast, ovarian, colorectal, uterine/endometrial, pancreatic, and melanoma). PV frequencies were highest among patients with ovarian cancer (13.8%) and lowest among patients with melanoma (8.1%). Fewer than half of PVs identified in patients meeting testing criteria for only *BRCA1/2* or only Lynch syndrome occurred in the respective genes (33.1% and 46.2%). In addition, 5.8% of patients with PVs in *BRCA1/2* and 26.9% of patients with PVs in Lynch syndrome genes did not meet respective testing criteria.

**Conclusion:**

Opportunities to improve upon identification of patients at risk for hereditary cancer predisposition include revising *BRCA1/2* and Lynch syndrome testing criteria to include additional clinically actionable genes with overlapping phenotypes and relaxing testing criteria for associated cancers.

## INTRODUCTION

Identification of patients at risk for inherited cancer susceptibility is dependent upon the ability to characterize genes and alterations associated with increased cancer risk and establish appropriate indications for genetic testing. With the exponential uptake of next-generation sequencing–based hereditary cancer panels, the curation and analysis of large data sets have furthered our understanding of a full spectrum of clinically relevant cancer predisposition genes. Efforts have included reinterpreting the phenotypic spectra of well-characterized hereditary cancer predisposition syndromes,^1–5^ defining high and moderate cancer risk genes through case–control^6–9^ and pedigree analysis-based^10^ approaches, and exome/genome sequencing–based investigation for novel gene discovery.^11–13^

In response, genetic testing guidelines have evolved over the past few years to incorporate genes included on multigene panel testing (MGPT) for hereditary cancer into clinical practice. For example, National Comprehensive Cancer Network (NCCN) Clinical Practice Guidelines in Oncology (NCCN Guidelines®) for Genetic/Familial High-Risk Assessment: Breast and Ovarian^14^ and NCCN Guidelines® for Genetic/Familial High-Risk Assessment: Colorectal^15^ provide some information surrounding cancer risks and management recommendations for a range of genes included on multigene panel tests. While much progress has been made with understanding the clinical relevance and implications of these genes, testing criteria remain limited to genes associated with historically established cancer syndromes such as *BRCA1/2*, *TP53*, and mismatch repair genes. Clinician preference for and utilization of broader panels of genes continue to increase despite the lack of explicit testing criteria for genes more recently associated with cancer risk and for cancers falling outside the traditional phenotypic spectra for established hereditary syndromes. As a consequence, medical policy coverage has been highly variable across health plans.^16–19^ While several studies and laboratories have reported on pathogenic variant (PV) detection rates across a spectrum of cancers and genes, and others have explored the sensitivity of various genetic testing criteria,^20–22^ an in-depth review of the performance of existing genetic testing criteria at genotypic and phenotypic levels is needed to help guide evidence-based criteria for genetic testing and clinical decision making.

In this retrospective review of clinical histories and molecular results from 165,000 patients undergoing hereditary cancer predisposition testing, we evaluated gene-specific associations with six different cancer types (breast, ovarian, colorectal, uterine/endometrial, pancreatic, and melanoma) and assessed the performance of NCCN genetic testing criteria for *BRCA*-related breast and/or ovarian cancer syndrome^14^ and Lynch syndrome.^15^ Together, these findings further inform the clinical approach to hereditary genetic testing across a range of cancer types.

## MATERIALS AND METHODS

### Study population

Study subjects included patients who underwent MGPT encompassing breast, ovarian, colorectal, uterine/endometrial, pancreatic, and pan-cancer indications between March 2012 and December 2016 at a single diagnostic laboratory (Ambry Genetics, Aliso Viejo, CA). Clinical histories were obtained from clinician-completed test requisition forms (TRFs) and from clinical documentation such as pedigrees and chart notes when provided. Case selection was limited to one individual per family. In the instance where multiple individuals from the same family underwent MGPT, the first family member to undergo panel testing was selected for inclusion in this study. In addition, individuals with clinical history suggestive of inherited colorectal polyposis, defined as ten or more colorectal adenomas or ten or more nonadenomatous colorectal polyps, or any number of hamartomatous polyps, were excluded from analysis. This study was deemed exempt from review by the Western Institutional Review Board.

### Multigene panel testing

Patients underwent comprehensive germline analysis of 5–49 genes depending on the multigene panel ordered (Table S1). With the exception of *GREM1* and *EPCAM*, Sanger or next-generation sequencing analysis was performed for all coding domains and well into the flanking 5’ and 3’ ends of all the introns and untranslated regions. With the exception of *GREM1*, *EPCAM*, and *MITF*, gross deletion/duplication analysis was performed for all covered exons and untranslated regions. Of note, the *APC* promoter 1B region was covered as part of deletion/duplication analysis. For *GREM1*, only the status of the 40-kb 5’UTR gross duplication was analyzed and reported and for *EPCAM*, only gross deletions encompassing the 3’ end of the gene were reported.

All variants, with the exception of previously characterized benign alterations, underwent thorough assessment and review of available evidence (e.g., population frequency information, published case reports, case–control and functional studies, internal co-occurrence and cosegregation data, evolutionary conservation, and in silico predictions). Variants were further classified per Ambry’s five-tier variant classification protocol (pathogenic mutation; variant, likely pathogenic [VLP]; variant of unknown significance [VUS]; variant, likely benign [VLB]; and benign) which is based on published recommendations/guidelines by the American College of Medical Genetics and Genomics and the International Agency for Research on Cancer.^23–25^ All identified alterations were deposited in ClinVar.

### Data analysis

Gene-specific pathogenic variant/VLP (herein collectively referred to as PVs) and VUS frequencies were assessed across a wide range of cancer phenotypic groups corresponding to *BRCA1/2* and Lynch syndrome testing criteria, along with the more common proband cancer types and combinations observed in this cohort. PV and VUS frequencies were based on variant classification as of December 2016. Analyses were primarily limited to 32 cancer susceptibility genes: *APC*, *ATM*, *BARD1*, *BRCA1*, *BRCA2*, *BRIP1*, *BMPR1A*, *CDH1*, *CDK4*, *CDKN2A*, *CHEK2*, *EPCAM*, *GREM1*, *MLH1*, *MRE11A*, *MSH2*, *MSH6*, *MUTYH*, *NBN*, *NF1*, *PALB2*, *PMS2*, *POLD1*, *POLE*, *PTEN*, *RAD50*, *RAD51C*, *RAD51D*, *SMAD4*, *SMARCA4*, *STK11*, *TP53*. *MUTYH* PV frequencies included biallelic carriers only. For ease of analysis and interpretation, pooled PV frequencies were often calculated for the following groups of genes: *BRCA1/2*, other breast and/or ovarian cancer genes (*ATM*, *CHEK2*, *PALB2*, *BARD1*, *BRIP1*, *RAD51C*, *RAD51D*, *NBN*, *TP53*, *CDH1*, *PTEN*, *NF1*), Lynch syndrome genes (*MLH1*, *EPCAM/MSH2*, *MSH6*, *PMS2*), and other cancer predisposition genes (*STK11*, *CDKN2A*, *CDK4*, *SMARCA4*, *APC*, *MUTYH*, *BMPR1A*, *SMAD4*, *GREM1*, *POLD1*, *POLE*, *MRE11A*, *RAD50*).

Case–control analysis was performed by pooling PVs to the gene level and comparing the frequency in Caucasian MGPT cases relative to non-Finnish European gnomAD exome controls from release 2.0.2 (*n* = 55,860).^26,27^ The frequency in gnomAD was restricted to PASS-only PVs, and if a variant was non-PASS in gnomAD and seen in the cancer case it was excluded from the frequency calculation. The odds ratio (OR) and a corresponding 95% confidence interval derived by inverting Fisher’s exact test^28^ were estimated for cases with a personal history limited to one of the six cancer types studied. Copy-number variants and large structural rearrangements identified in the cases were excluded from the frequency calculation to be consistent with gnomAD frequencies. All tests were two-sided and a *p* value less than 0.05 was considered statistically significant.

For all patients with personal and/or family history information provided, clinical histories were systematically evaluated to determine whether NCCN genetic testing criteria for *BRCA*-related breast and/or ovarian cancer syndrome^14^ and Lynch syndrome^15^ were met. Coded algorithms were generated for each criterion (e.g., breast cancer ≤45, colorectal cancer <50) and applied to curated clinical history information using Excel to generate a binary output indicating whether the patient passed or failed each criterion. A complete listing of each criterion assessed, including any exceptions and interpretations, is presented in Table S2 and Table S3 for *BRCA1/2* and Lynch syndrome, respectively. For each criterion, a subset of cases was manually reviewed to ensure accurate code output. Patients met testing criteria for a given syndrome if they passed at least one criterion for that syndrome, whereas those failing all criteria for a given syndrome did not meet the respective testing criteria. A subset of cases was independently reviewed by a certified genetic counselor, and the output of the coding algorithm accurately identified patients as meeting or not meeting criteria in all cases.

## RESULTS

### Characteristics of the multigene panel testing cohort

Demographics and clinical characteristics of the MGPT cohort are shown in Table 1. Patients were predominantly female (94.2%) and the median (interquartile range) age at testing was 52 (43, 62) years. The most frequent self-reported race/ethnicity was Caucasian (64.0%), followed by African American (6.5%), Ashkenazi Jewish (6.1%), Hispanic (6.0%), and Asian (4.2%). A vast majority of patients (89.8%) met NCCN testing criteria for *BRCA1/2* (83.9%) and/or Lynch syndrome (20.3%). Most patients were reported to have a personal history of cancer (72.5%), approximately half of which was female breast cancer (54.4%). Ovarian (7.6%), colorectal (5.4%), uterine/endometrial (3.3%), pancreatic cancer (1.2%), and melanoma (2.0%) were the more commonly reported tumor types among the remaining cancers. Additional observed cancers are in Table S4. Overall, 13.3% of patients with cancer were reported to have a history of multiple cancer types; however, this varied widely in the context of specific cancers (Table S4). Most patients also reported a family history of cancer among first- and second-degree relatives (90.1%), with breast (59.8%), colorectal (26.0%), and ovarian (17.8%) cancers among the most commonly reported (Table 1).

**Table 1:** Demographics and clinical history of multigene cancer panel cohort (*n* = 165,024)

Characteristic	*n*	%	Characteristic	*n*	%
Gender			Proband history		
Male	9616	5.8	Personal history of cancer	119,665	72.5
Female	155,406	94.2	Multiple cancer types^b^	15,965	13.3
Unknown	2	0.0	Breast (female)	89,225	54.4
Age at testing			Multiple primary breast (female)^c^	12,077	13.5
Median (IQR)	52 (43, 62)		Ovarian	12,602	7.6
Ethnicity			Melanoma	3360	2.0
African American/Black	10,659	6.5	Pancreatic	2046	1.2
Ashkenazi Jewish	10,143	6.1	Colorectal	8907	5.4
Asian	6949	4.2	Endometrial	5435	3.3
Caucasian	105,582	64.0	No personal history of cancer	40,594	24.6
Hispanic	9845	6.0	Not provided	4765	2.9
Mixed ethnicity/other	10,619	6.4	Family history (1st and 2nd degree relatives)		
Unknown	11,227	6.8	Any	148,666	90.1
Clinical history provided			Breast	98,743	59.8
Personal and/or family history provided	163,877	99.3	Ovarian	29,296	17.8
Personal and family history left blank	1147	0.7	Colorectal	42,926	26.0
Testing criteria^a^			Endometrial	13,759	8.3
Meet NCCN *BRCA1/2* criteria	137,446	83.9	Pancreatic	17,953	10.9
Meet NCCN Lynch syndrome criteria	33,278	20.3	Melanoma	7970	4.8
Meet both criteria	23,632	14.4	Prostate	31,711	19.2
Meet neither criteria	16,785	10.2	No family history of cancer	8830	5.4
			Family history not provided	7528	4.6

*IQR* interquartile range, *NCCN* National Comprehensive Cancer Network.

^a^Percent of 163,877 patients with personal and/or family history provided.

^b^Percent of 119,665 patients with cancer.

^c^Percent of 89,255 female breast cancer patients.

BreastNext (a 17-gene breast cancer panel) was the most frequently ordered panel overall (23.8%); however, starting in 2015 and throughout the remainder of the study time period, CancerNext (a 34-gene pan-cancer panel) was the most frequently ordered panel (28.2% in the last quarter of 2016) (Figure S1). PV detection rates ranged from 5.5% on the smallest cancer panel to 10.8% on the largest cancer panel, with up to 3.3% of PV carriers identified to carry PVs in ≥2 genes tested (Table S1). Detection rates for VUS similarly corresponded to cancer panel size, with 5.4% to 39.5% of patients identified to carry at least one VUS. PVs identified via gross deletion/duplication (del/dup) analysis accounted for 8.3% of PVs detected, though this varied widely by gene (Table 2, Table S5). For example, among the mismatch repair genes, gross del/dups accounted for 2.2% (*MSH6*) to 28.5% (*MSH2*) of PVs.

**Table 2:** Percentage of pathogenic/likely pathogenic variants identified via gross deletion/duplication analysis

Gene	Overall		
	Gross del/dup, *n*	Total PV, *n*	% of PVs
*APC*	6	100	6.0%
*ATM*	64	1384	4.6%
*BARD1*	26	236	11.0%
*BRCA1*	337	2555	13.2%
*BRCA2*	73	2681	2.7%
*BRIP1*	23	400	5.8%
*BMPR1A*	2	15	13.3%
*CDH1*	9	110	8.2%
*CDKN2A*	4	146	2.7%
*CHEK2*	137	2114	6.5%
*EPCAM*^a^	21	21	100.0%
*GREM1*	5	5	100.0%
*MLH1*	36	308	11.7%
*MRE11A*	9	140	6.4%
*MSH2*	103	362	28.5%
*MSH6*	10	446	2.2%
*MUTYH*	2	53	3.8%
*NBN*	15	264	5.7%
*NF1*	8	139	5.8%
*PALB2*	83	921	9.0%
*PMS2*	108	407	26.5%
*POLD1*	0	1	0.0%
*POLE*	0	0	n/a
*PTEN*	8	105	7.6%
*RAD50*	4	328	1.2%
*RAD51C*	44	271	16.2%
*RAD51D*	12	140	8.6%
*SMAD4*	0	17	0.0%
*SMARCA4*	1	2	50.0%
*STK11*	1	3	33.3%
*TP53*	13	303	4.3%
Total	1164	13,977	8.3%

*PV* pathogenic variant.

^a^Includes 5 cases with deletion of *EPCAM* only and 16 cases with a deletion involving *EPCAM* and *MSH2.*

### Associated cancer risks

**Fig. 1:**
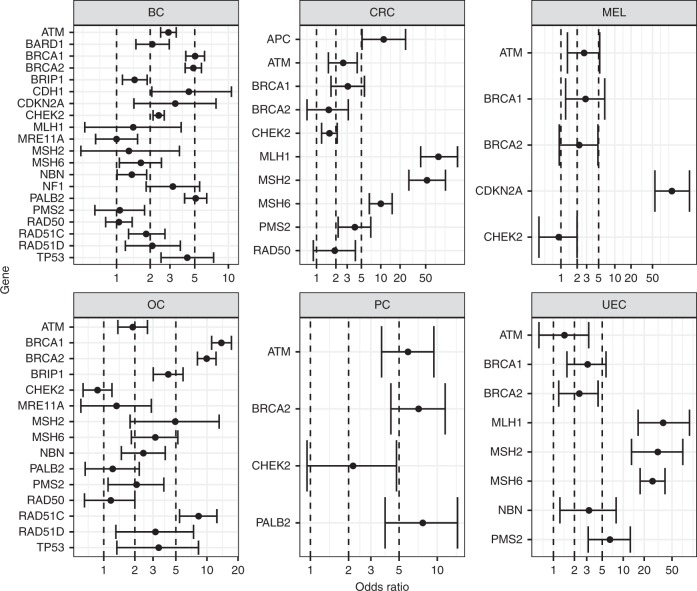
**Gene-specific cancer associations derived from panel cases vs. gnomAD non-Finnish European (NFE) reference controls.** Gene-specific associations, along with corresponding 95% confidence intervals (CI), are shown for breast cancer (BC), colorectal cancer (CRC), melanoma (MEL), ovarian cancer (OC), pancreatic cancer (PC), and uterine/endometrial cancer (UEC). Case–control analysis was performed by pooling pathogenic variants (PVs) to the gene level and comparing the frequency in Caucasian multigene panel testing (MGPT) cases relative to NFE gnomAD controls. Genes with <5 PVs detected in cases and/or controls are not shown; however, complete analysis results are shown in Table S4.

Gene-specific cancer risks were estimated by comparing PV frequencies among Caucasian cancer cases to non-Finnish European reference controls from gnomAD (Fig. 1, Table S6). Genes with PVs demonstrating statistically significant associations with breast cancer included *BRCA1/2*, *ATM*, *CHEK2*, *PALB2*, *BARD1*, *BRIP1*, *RAD51C*, *RAD51D*, *NBN*, *CDH1*, *NF1*, *TP53*, *CDKN2A,* and *MSH6*. While PVs in most of these genes were associated with two- to fivefold increased risks of breast cancer, several genes were significantly associated with increased breast cancer risk <twofold (*BRIP1*, *MSH6*, *NBN*, and *RAD51C*). PVs in nine of these genes with elevated breast cancer risk were also associated with increased risk for ovarian cancer (*BRCA1/2*, *ATM*, *BRIP1*, *RAD51C/D*, *NBN*, *TP53*, and *MSH6)*, along with *MSH2* and *PMS2*. Odds ratios for ovarian cancer across these 11 genes ranged from 1.91 for *ATM* to 13.8 for *BRCA1*. PVs in *BRCA2*, *PALB2*, and *ATM* were significantly associated with increased risk for pancreatic cancer, and PVs in *BRCA1*, *ATM*, and *CDKN2A* were associated with increased risk for melanoma. As expected, PVs in *MLH1*, *MSH2*, *MSH6*, *PMS2*, and *APC* were significantly associated with increased risk for colorectal cancer. PVs in *CHEK2*, *ATM*, and *BRCA1* were also associated with increased risk for colorectal cancer; however, risks were moderate in comparison with the Lynch syndrome genes and *APC*. PVs in *MLH1*, *MSH2*, *MSH6*, and *PMS2* were also expectedly associated with increased risk for uterine/endometrial cancer. In addition, PVs in *BRCA1, BRCA2*, and *NBN* were significantly associated with moderately increased risks for uterine/endometrial cancer. PVs in *MRE11A* and *RAD50* were not significantly associated with increased risks for any cancer types studied and thus were excluded from PV frequency calculations. The following sensitivity analyses were performed for each cancer type: all patients with the respective cancer types, patients with each respective cancer as their first cancer, patients with no prior genetic testing reported, and patients with the respective cancer as their first and only cancer with no prior genetic testing reported. With minor exceptions, risk estimates were highly similar across all sensitivity analyses.

### Pathogenic/likely pathogenic variant frequency by gene and clinical history

**Fig. 2:**
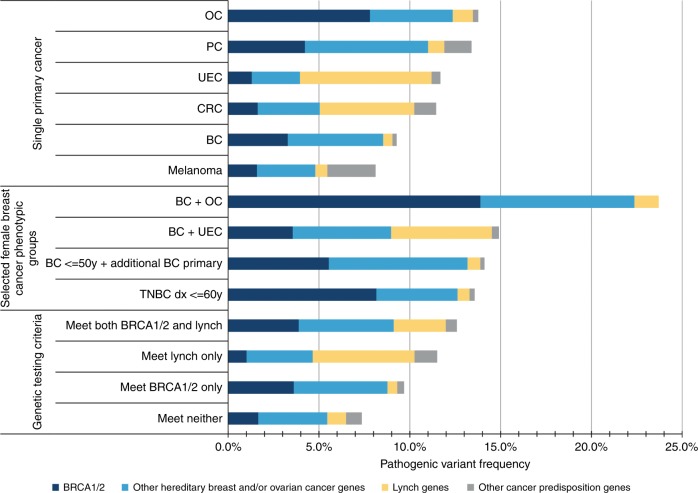
**Pathogenic variant frequency by gene and clinical history.** Pooled pathogenic variant frequencies (%) are shown across a range of phenotypic groups based on proband and family history of cancer. Genes were grouped as follows: *BRCA1/2*, other breast and/or ovarian cancer genes (*ATM*, *CHEK2*, *PALB2*, *BARD1*, *BRIP1*, *RAD51C*, *RAD51D*, *NBN*, *TP53*, *CDH1*, *PTEN*, *NF1*), Lynch syndrome genes (*MLH1*, *EPCAM/MSH2*, *MSH6*, *PMS2*), and other cancer predisposition genes (*STK11*, *CDKN2A*, *CDK4*, *SMARCA4*, *APC*, *MUTYH*, *BMPR1A*, *SMAD4*, *GREM1*, *POLD1*, *POLE*). Pooled pathogenic frequencies exclude patients with additional cancer types unless otherwise noted. With the exception of “BC <50 + additional primary BC”, pooled pathogenic frequencies are limited to females with a single primary breast cancer. *BC* breast cancer, *CRC* colorectal cancer, *OC* ovarian cancer, *PC* pancreatic cancer, *TNBC* triple negative breast cancer, *UEC* uterine/endometrial cancer.

PV frequencies for a range of cancer phenotypic groups are shown in Fig. 2 and Table S7. Among the six cancer types studied, PVs were most frequent among patients with ovarian cancer and least frequent among patients with melanoma. PV distribution varied between and within cancer phenotypic groups. For example, among breast cancer patients, cumulative PV frequencies were highest for patients also reporting a history of ovarian cancer (23.7%), uterine/endometrial cancer (14.9%), breast cancer <50 with an additional breast cancer primary (14.1%), and triple negative breast cancer (TNBC) ≤60 (13.6%) (Fig. 2, Table S7). Among women with breast and ovarian cancer or TNBC ≤60, PVs in *BRCA1/2* were more frequent than PVs in other breast and/or ovarian cancer genes, whereas PVs in other breast and/or ovarian cancer genes were more frequent than *BRCA1/2* among women with breast and uterine/endometrial cancer or breast cancer <50 and additional breast cancer primary. Among patients meeting testing criteria (*BRCA1/2*, Lynch, or both), the combined PV frequency increased when a multigene panel was ordered compared with when only the indicated genes were analyzed (Fig. 2, Table S7).

### Performance of *BRCA1/2* and Lynch syndrome testing criteria

*BRCA1/2* testing criteria expectedly yielded the highest sensitivity for these genes, with 94.2% of *BRCA1/2* PV carriers meeting criteria (Table S8). Interestingly, over half (59.1%, *n* = 143/242) of the female *BRCA1/2* PV carriers not meeting *BRCA1/2* testing criteria had a personal history of breast cancer. Further, 33.6% (*n* = 48) of these PV carriers reported breast cancer under age 50, 7.0% (*n* = 10) reported multiple primary breast cancers, and 6.3% (*n* = 9) reported triple negative breast cancers. In contrast to *BRCA1/2*, only 73.1% of mismatch repair genes/*EPCAM* PV carriers met Lynch criteria. Sensitivity was highest for *MLH1* (89.3%) and lowest for *PMS2* (52.0%) (Table S8). In fact, these criteria yielded higher sensitivity for *MUTYH* (biallelic) and *APC* PV carriers than for *PMS2*. Of patients with PVs in Lynch syndrome genes failing to meet Lynch criteria, 41.5% had a personal history of a Lynch syndrome–related cancer.

**Fig. 3:**
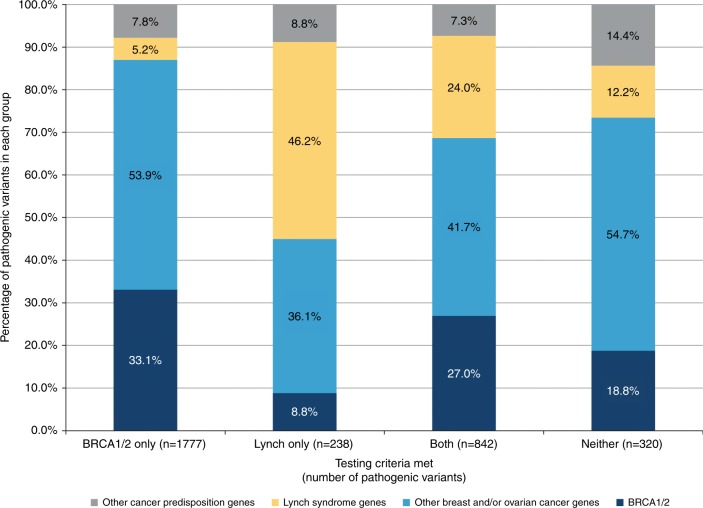
**Distribution of pathogenic/likely pathogenic variants based on genetic testing criteria met.** The distribution of pathogenic variants (PVs) based on genetic testing criteria met was assessed for patients tested for all 32 cancer predisposition genes (*n* = 33,987). Total PV counts among patients meeting only *BRCA1/2* criteria, only Lynch criteria, both criteria, and neither criteria are shown in parenthesis. Genes were grouped as follows: *BRCA1/2*, other breast and/or ovarian cancer genes (*ATM*, *CHEK2*, *PALB2*, *BARD1*, *BRIP1*, *RAD51C*, *RAD51D*, *NBN*, *TP53*, *CDH1*, *PTEN*, *NF1*), Lynch syndrome genes (*MLH1*, *EPCAM/MSH2*, *MSH6*, *PMS2*), and other cancer predisposition genes (*STK11*, *CDKN2A*, *CDK4*, *SMARCA4*, *APC*, *MUTYH*, *BMPR1A*, *SMAD4*, *GREM1*, *POLD1*, *POLE*, *MRE11A*, *RAD50*).

In an assessment of patients tested for all 32 cancer predisposition genes (*n* = 33,987), less than half of PVs in patients meeting criteria for only *BRCA1/2* or only Lynch syndrome occurred in the respective genes (33.1% and 46.2%, respectively) (Fig. 3). Among patients meeting criteria for only *BRCA1/2*, 53.9% of PVs occurred in other breast and/or ovarian cancer genes, 5.2% in Lynch syndrome genes, and 7.8% in other cancer predisposition genes. Among those meeting criteria for only Lynch syndrome, 8.8% of PVs occurred in *BRCA1/2*, 36.1% in other breast and/or ovarian cancer genes, and 8.8% in other cancer predisposition genes. Clinical histories of patients with PVs who did not meet either of these criteria are summarized in Table S9.

## DISCUSSION

In response to substantial data published on the increased efficacy of MGPT, most cancer geneticists have evolved their testing practices, yet the optimal testing strategy remains to be determined. In this study, we systematically evaluated the association of 32 cancer predisposition genes across multiple common phenotypes referred for MGPT with regard to PV frequency, associated cancer risks, and proportion meeting genetic testing criteria for *BRCA1/2*-related hereditary breast and ovarian cancer and Lynch syndrome to better inform clinical multigene panel selection, as well as medical policy at a broader level.

Results from this study and others have confirmed substantial genotypic heterogeneity surrounding predisposition to cancers commonly referred for germline genetic testing. Consequently, *BRCA1/2* and Lynch syndrome testing criteria are not highly specific to these genes. In this study, 67% of PVs in patients meeting criteria for only *BRCA1/2* occurred in genes other than *BRCA1/2*, including 5.2% in Lynch syndrome genes (Fig. 3). Likewise, more than half (53.8%) of PVs in patients meeting only Lynch criteria were in non–Lynch syndrome genes, including 8.8% in *BRCA1/2* (Fig. 3). Similar findings were observed in another large laboratory-based study, though overall PV detection rates were lower due to a larger proportion of unaffected patients.^20^ Together, these findings support the revision of *BRCA1/2* and Lynch syndrome testing criteria to include a broader spectrum of cancer predisposition genes, particularly those with consensus management recommendations and overlapping phenotypes.

Our data also support even broader use of larger panels that contain a variety of hereditary cancer genes, even when patients lack classic clinical features associated with some of the genes, as evidenced by the mismatch in testing criteria met with what genes were actually mutated in some patients. For example, a significant proportion of those patients (20.0%) who tested positive for a PV in one of the mismatch repair genes met testing criteria for *BRCA1/2* alone, including 37.4% of *PMS2* PV carriers. In total, 409 Lynch syndrome diagnoses would have gone undetected had more targeted testing been ordered in this cohort alone, resulting in missed opportunities for patients to pursue interventions known to reduce risk and decrease mortality.^29–31^

We also observed a subset of patients with PVs in *BRCA1/2* who did not meet testing criteria for these genes (5.8%), suggesting a need to revise testing criteria to identify more at-risk patients. Beitsch et al. recently reported on the yield of an 80-gene panel among 1001 breast cancer patients, comparing those meeting versus not meeting NCCN criteria and reporting no significant difference in PV rates (9.39% and 7.92%, respectively).^22^ However, PV rates included all genes tested regardless of association with breast cancer or clinical actionability; characteristics of PV carriers with breast cancer not meeting NCCN criteria, such as age at diagnosis, tumor pathology, and family history of related cancers were not considered; and the data were based on a 2017 version of the guideline. Despite these limitations, the study by Beitsch et al. demonstrates the need for revisions to genetic testing guidelines for breast cancer.

Findings from this study also demonstrate the importance of including comprehensive assessment for gross del/dups as part of hereditary cancer MGPT. The prevalence of gross del/dups has not previously been well described, with the exception of *BRCA1/2* and Lynch syndrome genes.^32,33^ For a number of genes, del/dups accounted for greater than 10% of PVs, indicating that a substantial proportion of at-risk patients would be missed if del/dup testing methods are not included in MGPT.

Although we have previously reported on gene-specific risks for breast,^6^ ovarian,^34^ and pancreatic^35^ cancer, updated analyses were conducted due to the availability of additional multigene panel cases (Ambry Genetics) and reference controls (gnomAD). Results were highly consistent with initial reports, with some minor exceptions, which are likely attributable to a much larger sample size in the current study, differences in the inclusion criteria applied to cases, and different quality control metrics applied to ExAC versus gnomAD controls. For example, *CDKN2A*, *NF1*, and *NBN* were associated with increased risk for breast cancer in the current study, but were not significant in previously published analyses in our smaller cohorts. The association of germline *CDKN2A* PVs with increased breast cancer risk is of particular importance because, to our knowledge, this has not been reported previously.

Risk estimates were also generated for colorectal and uterine/endometrial cancers and melanoma, with this being the first time colorectal and uterine/endometrial cancer risk estimates are presented for mismatch repair genes in a MGPT cohort. As expected, PVs in *MLH1*, *MSH2*, *MSH6*, and *PMS2* were significantly associated with increased risks for colorectal and uterine/endometrial cancers. Further, our findings are consistent with current understanding of the cancer risk profile for each gene: colorectal and uterine/endometrial cancer risks were highest for *MLH1* and *MSH2* and lower for *MSH6* and *PMS2*. PVs in *MSH2*, *MSH6*, and *PMS2* were significantly associated with increased risk for ovarian cancer, while the number of PVs in *MLH1* was too small to reliably assess for an association. The findings in *MSH6* and *PMS2* are of particular clinical importance, as these data are extremely limited in the literature until recently, yet clinicians counsel about a general increased risk for ovarian cancer with PVs in Lynch syndrome genes. Thus, having evidence-based data to support these clinical claims is of huge importance. PVs in *APC*, *CDH1*, *CHEK2*, and *TP53* also showed significant associations with increased colorectal cancer risk. *ATM* and *BRCA1* were unexpectedly associated with moderately increased risk for colorectal cancer in this study, with 20.7% and 19.4% of PV carriers in these genes meeting testing criteria for Lynch syndrome (Table S8). While these genes have not traditionally been considered as colorectal cancer susceptibility genes, associations have been reported for *BRCA1* and *ATM*.^36–38^ *BRCA1* and *BRCA2* were also associated with increased risks for uterine/endometrial cancer in this study. Regarding melanoma, while risk estimates were not reliable for most genes due to a smaller melanoma cohort size, PVs in *CDKN2A* were expectedly associated with very high risks for melanoma (OR = 114.6); however, PVs in *ATM* and *BRCA1* were also elevated (two- to threefold increased risks).

While recommendations for increased surveillance and/or surgical implications are already in place for most genes conferring significantly increased cancer risks, results from this study revealed several additional gene–cancer associations that have the potential to impact clinical management for PV carriers if replicated in other studies. For example, PVs in *ATM* and *BRCA1* were significantly associated with >twofold increased risk for colorectal cancer and PVs in *RAD51D* and *CDKN2A* were significantly associated with >twofold increased risk for breast cancer. At these levels of risk, consideration might be given to recommending more frequent colonoscopy or addition of breast magnetic resonance imaging (MRI); however, these findings (and others) should be replicated in well-matched case–control studies and/or pedigree-based penetrance analyses before any changes are made to clinical practice for PV carriers. In particular, studies involving age-matched controls are needed to assess age-specific cancer risks to inform the age at which to implement various screening and surgical interventions.

PVs in *MRE11A* and *RAD50* were not significantly associated with increased risks for any of the cancers investigated in this study. These genes were initially proposed as candidate breast cancer genes based on their role in double strand break repair via the MRN protein complex. While results from this study do not support the routine inclusion of these genes in multigene hereditary cancer panels, additional studies focused on other types of alterations such as gross del/dups and rare missense VUS are needed to fully appreciate any role these genes may play in inherited cancer risk.

Several potential limitations should be noted in addition to those previously mentioned. First, clinical history information was primarily obtained from clinician-completed TRFs, although 39% of cases did include documentation directly from clinic notes. Though it is possible that pertinent cancer history was either not reported or incorrectly reported on the TRF, results from a recent study found that TRF-based cancer history from the laboratory used in this study—specifically cancer type and age at diagnosis—is of high quality for probands and their close relatives.^39^ However, more detailed clinical history information than what was routinely available in this study is sometimes necessary to fully apply NCCN testing criteria. For example, Gleason score is needed to fully assess *BRCA1/2* testing criteria; however, this was not systematically included on the TRF and thus unavailable for many prostate cancer patients in this study. Another important consideration in the interpretation of these findings is that the PV detection rates and cancer risk analysis were primarily based on patient cancer history. While we attempted to minimize potential confounding effects introduced by other cancers, we did not control for family history in these analyses, although family history was incorporated where applicable when assessing *BRCA1/2* and Lynch syndrome testing criteria. Lastly, despite this being the largest cohort reported to date for breast, ovarian, uterine/endometrial, colorectal cancers, and melanoma, risk estimates remain limited by size for cancers other than breast, and cancer risk analysis was not informative for many genes due to low PV count and rarity of PVs in many of these genes. Limitations also exist surrounding the cohorts used for case–control analysis, as previously described by our group.^6^

Results from this study collectively highlight the extensive genetic heterogeneity surrounding cancers commonly referred for germline testing, along with the extensive overlap in cancer phenotypes associated with cancer predisposition genes. These findings affirm the clinical validity of a multigene panel testing approach involving comprehensive evaluation of genes for patients with a wide spectrum of cancer histories. Opportunities to improve identification of patients at risk include revising *BRCA1/2* and Lynch syndrome testing criteria to include a broader range of medically actionable cancer predisposition genes and relaxing criteria, such as age at diagnosis constraints, for patients with associated cancers. Such revision of clinical genetic testing guidelines will be more aligned with clinician practice and will better guide health plan medical policy, ultimately improving patient access to testing.

## Supplementary information

Supplementary Table S1

Supplementary Table S2

Supplementary Table S3

Supplementary Table S4

Supplementary Table S5

Supplementary Table S6

Supplementary Table S7

Supplementary Table S8

Supplementary Table S9

Supplementary Figure S1

## References

[1] Espenschied CR, LaDuca H, Li S, McFarland R, Gau CL, Hampel H (2017). Multigene panel testing provides a new perspective on Lynch syndrome. J Clin Oncol..

[2] Lowstuter K, Espenschied CR, Sturgeon D (2017). Unexpected CDH1 mutations identified on multigene panels pose clinical management challenges. JCO Precis Oncol..

[3] Rana HQ, Gelman R, LaDuca H (2018). Differences in TP53 mutation carrier phenotypes emerge from panel-based testing. J Natl Cancer Inst..

[4] Roberts ME, Jackson SA, Susswein LR (2018). MSH6 and PMS2 germ-line pathogenic variants implicated in Lynch syndrome are associated with breast cancer. Genet Med..

[5] Susswein LR, Marshall ML, Nusbaum R (2016). Pathogenic and likely pathogenic variant prevalence among the first 10,000 patients referred for next-generation cancer panel testing. Genet Med..

[6] Couch FJ, Shimelis H, Hu C (2017). Associations between cancer predisposition testing panel genes and breast cancer. JAMA Oncol..

[7] Hu C, Hart SN, Polley EC (2018). Association between inherited germline mutations in cancer predisposition genes and risk of pancreatic cancer. JAMA..

[8] Kurian AW, Hughes E, Handorf EA, et al. Breast and ovarian cancer penetrance estimates derived from germline multiple-gene sequencing results in women. JCO Precis Oncol. 2017:1–12. https://ascopubs.org/doi/full/10.1200/PO.16.00066. Accessed 9 August 2019. 10.1200/PO.16.00066 35172496

[9] Norquist BM, Harrell MI, Brady MF (2016). Inherited mutations in women with ovarian carcinoma. JAMA Oncol..

[10] Antoniou AC, Foulkes WD, Tischkowitz M (2014). Breast-cancer risk in families with mutations in PALB2. N Engl J Med..

[11] Palles C, Cazier JB, Howarth KM (2013). Germline mutations affecting the proofreading domains of POLE and POLD1 predispose to colorectal adenomas and carcinomas. Nat Genet..

[12] Roberts NJ, Jiao Y, Yu J (2012). ATM mutations in patients with hereditary pancreatic cancer. Cancer Discov..

[13] Witkowski L, Carrot-Zhang J, Albrecht S (2014). Germline and somatic SMARCA4 mutations characterize small cell carcinoma of the ovary, hypercalcemic type. Nat Genet..

[14] National Comprehensive Cancer Network. NCCN Clinical Practice Guidelines in Oncology (NCCN Guidelines®) for genetic/familial high-risk assessment: breast and ovarian V.1. 2018. https://www.nccn.org/professionals/physician_gls/default.aspx#detection. Accessed 11 July 2018.

[15] National Comprehensive Cancer Network. NCCN Clinical Practice Guidelines in Oncology (NCCN Guidelines®) for genetic/familial high-risk assessment: colorectal V.1. 2018. https://www.nccn.org/professionals/physician_gls/default.aspx#detection. Accessed 12 July 2018. 10.6004/jnccn.2016.0108 27496117

[16] Aetna. Genetic testing. 2018. http://www.aetna.com/cpb/medical/data/100_199/0140.html. Accessed 9 August 2019.

[17] Cigna. Genetic testing for hereditary cancer susceptibility syndromes. 2018. https://cignaforhcp.cigna.com/public/content/pdf/coveragePolicies/medical/mm_0518_coveragepositioncriteria_genetic_cancer_syndromes.pdf. Accessed 9 August 2019.

[18] United Healthcare. Genetic testing for hereditary cancer. Medical policy. 2019. https://www.uhcprovider.com/content/dam/provider/docs/public/policies/comm-medical-drug/genetic-testing-hereditary-breast-ovarian-cancer-syndrome.pdf. Accessed 9 August 2019.

[19] Humana. Genetic testing for cancer susceptibility. 2018. http://apps.humana.com/tad/tad_new/home.aspx. Accessed 9 August 2019.

[20] Rosenthal ET, Bernhisel R, Brown K, Kidd J, Manley S (2017). Clinical testing with a panel of 25 genes associated with increased cancer risk results in a significant increase in clinically significant findings across a broad range of cancer histories. Cancer Genet..

[21] Yang S, Axilbund JE, O’Leary E (2018). Underdiagnosis of hereditary breast and ovarian cancer in Medicare patients: genetic testing criteria miss the mark. Ann Surg Oncol.

[22] Beitsch PD, Whitworth PW, Hughes K (2019). Underdiagnosis of hereditary breast cancer: are genetic testing guidelines a tool or an obstacle?. J Clin Oncol..

[23] Pesaran T, Karam R, Huether R (2016). Beyond DNA: an integrated and functional approach for classifying germline variants in breast cancer genes. Int J Breast Cancer..

[24] Plon SE, Eccles DM, Easton D (2008). Sequence variant classification and reporting: recommendations for improving the interpretation of cancer susceptibility genetic test results. Human Mutat..

[25] Richards S, Aziz N, Bale S (2015). Standards and guidelines for the interpretation of sequence variants: a joint consensus recommendation of the American College of Medical Genetics and Genomics and the Association for Molecular Pathology. Genet Med..

[26] Genome Aggregation Database (gnomAD). http://gnomad.broadinstitute.org/. Accessed 11 November 2016.

[27] Lek M, Karczewski KJ, Minikel EV (2016). Analysis of protein-coding genetic variation in 60,706 humans. Nature..

[28] Fay MP (2010). Confidence intervals that match Fisher’s exact or Blaker’s exact tests. Biostatistics..

[29] Jarvinen HJ, Aarnio M, Mustonen H (2000). Controlled 15-year trial on screening for colorectal cancer in families with hereditary nonpolyposis colorectal cancer. Gastroenterology..

[30] de Jong AE, Hendriks YM, Kleibeuker JH (2006). Decrease in mortality in Lynch syndrome families because of surveillance. Gastroenterology..

[31] Schmeler KM, Lynch HT, Chen LM (2006). Prophylactic surgery to reduce the risk of gynecologic cancers in the Lynch syndrome. N Engl J Med..

[32] Gylling A, Ridanpaa M, Vierimaa O (2009). Large genomic rearrangements and germline epimutations in Lynch syndrome. Int J Cancer..

[33] Judkins T, Rosenthal E, Arnell C (2012). Clinical significance of large rearrangements in BRCA1 and BRCA2. Cancer..

[34] Lilyquist J, LaDuca H, Polley E (2017). Frequency of mutations in a large series of clinically ascertained ovarian cancer cases tested on multigene panels compared to reference controls. Gynecol Oncol..

[35] Hu C, LaDuca H, Shimelis H (2018). Multigene hereditary cancer panels reveal high-risk pancreatic cancer susceptibility genes. JCO Precis Oncol..

[36] AlDubayan SH, Giannakis M, Moore ND (2018). Inherited DNA-repair defects in colorectal cancer. Am J Hum Genet..

[37] Phelan CM, Iqbal J, Lynch HT (2014). Incidence of colorectal cancer in BRCA1 and BRCA2 mutation carriers: results from a follow-up study. Br J Cancer..

[38] Thompson D, Duedal S, Kirner J (2005). Cancer risks and mortality in heterozygous ATM mutation carriers. J Natl Cancer Inst..

[39] LaDuca H, McFarland R, Gutierrez S (2018). Quality of clinician-reported cancer history when ordering genetic testing. JCO Clin Cancer Inform..

