# Supplementary material for: A clinical guide to hereditary cancer panel testing: evaluation of gene-specific cancer associations and sensitivity of genetic testing criteria in a cohort of 165,000 high-risk patients
Source: Genet Med. 2019 Aug 13;22(2):407–15. doi: 10.1038/s41436-019-0633-8 (PMC7000322; doi:10.1038/s41436-019-0633-8)
Supplement: Supplementary file 2 — Supplementary Table S2 [file 41436_2019_633_MOESM2_ESM.docx]

Table S2. Application of NCCN genetic testing criteria for BRCA-Related breast and/or ovarian cancer syndrome

| Criterion*^a^* | Description of exceptions and/or interpretations made in the application of criterion |
| --- | --- |
| Individual from a family with a known deleterious *BRCA1*/*BRCA2* gene mutation | NOT APPLIED - In this scenario it is recommended to perform genetic testing for the familial mutation. Since the study population was patients who underwent multigene panel testing (and not testing for a single mutation), we did not feel it was appropriate to apply or evaluate this criterion. Further, in the event this information is provided for a given case, nomenclature for the familial mutation may not be readily available, making it difficult to assess whether the mutation was truly pathogenic. |
| Breast cancer <=45y |  |
| Breast cancer <=50y and an additional breast cancer primary |  |
| Breast cancer <=50y and >=1 close blood relative*^b^* with breast cancer at any age |  |
| Breast cancer and >=1 close blood relative*^b^* with pancreatic cancer |  |
| Breast cancer and >=1 close blood relative*^b^* with high-grade (Gleason score >=7) or metastatic prostate cancer |  |
| Breast cancer <=50y and an unknown or limited family history | NOT APPLIED - NCCN provides the following example for unknown or limited family history/structure, “fewer than 2 female first- or second-degree relatives having lived beyond age 45 in either lineage”. This criterion could not reliably be assessed in this study population since pedigrees or other detailed description of family structure was not available for most patients. |
| Triple negative breast cancer <=60y |  |
| Breast cancer and >=2 additional diagnoses of breast cancer at any age in patient and/or in close blood relatives*^b^* |  |
| Breast cancer and >=1 close blood relatives*^b^* with breast cancer <=50y |  |
| Breast cancer and >=1 close blood relatives*^b^* with ovarian cancer |  |
| Breast cancer and a close male blood relative*^b^* with breast cancer |  |
| Breast cancer and of Ashkenazi Jewish ancestry |  |
| Ovarian cancer |  |
| Male breast cancer |  |
| Prostate cancer (Gleason score>=7) at any age with >=1 close blood relative*^b^* with ovarian cancer |  |
| Prostate cancer (Gleason score>=7) at any age with >=1 close blood relatives*^b^* with breast cancer <=50y |  |
| Prostate cancer (Gleason score>=7) at any age with >=2 close blood relatives*^b^* with breast cancer or prostate cancer (any grade) at any age |  |
| Prostate cancer (Gleason score>=7) at any age with >=1 close blood relative*^b^* with pancreatic cancer |  |
| Prostate cancer (Gleason score>=7) at any age with >=1 close blood relatives*^b^* with metastatic prostate cancer at any age |  |
| Metastatic prostate cancer |  |
| Pancreatic cancer |  |
| Prostate cancer (Gleason score>=7) at any age and Ashkenazi Jewish ancestry |  |
| *BRCA1/2* mutation detected by tumor profiling in the absence of germline mutation analysis | NOT APPLIED - Similar to the scenario of a known familial mutation in the family, patients would likely undergo confirmatory testing for a single variant in this scenario unless otherwise indicated based on other clinical history factors. Further, in the event this information is provided for a given case, nomenclature for the familial mutation may not be readily available, making it difficult to assess whether the mutation was truly pathogenic. |
| First or second-degree blood relative*^b^* meeting any of the above criteria*^c^*: |  |
| - Individual from a family with a known deleterious *BRCA1*/*BRCA2* gene mutation | NOT APPLIED - In this scenario it is recommended to perform genetic testing for the familial mutation. Since the study population was patients who underwent multigene panel testing (and not testing for a single mutation), we did not feel it was appropriate to apply or evaluate this criterion. Further, in the event this information is provided for a given case, nomenclature for the familial mutation may not be readily available, making it difficult to assess whether the mutation was truly pathogenic. |
| - Breast cancer <=45y | 1st or 2nd degree relative with breast cancer <=45 |
| - Breast cancer <=50y and an additional breast cancer primary | 1st or 2nd degree relative with breast cancer <=50 AND an additional breast cancer primary |
| - Breast cancer <=50y and >=1 close blood relative*^b^* with breast cancer at any age | 1st or 2nd degree relative with breast cancer <=50 and at least one additional 1st 2nd or 3rd degree relative on the same side of the family*^d^* with breast cancer |
| - Breast cancer and >=1 close blood relative*^b^* with pancreatic cancer | 1st or 2nd degree relative with breast cancer and at least one additional 1st 2nd or 3rd degree relative on the same side of the family*^d^* with pancreatic cancer |
| - Breast cancer and >=1 close blood relative*^b^* with high-grade (Gleason score >=7) or metastatic prostate cancer | 1st or 2nd degree relative with breast cancer and at least one additional 1st 2nd or 3rd degree relative on the same side of the family*^d^* with prostate cancer (Gleason >=7 or metastatic) |
| - Breast cancer <=50y and an unknown or limited family history | NOT APPLIED - NCCN provides the following example for unknown or limited family history/structure, “fewer than 2 female first- or second-degree relatives having lived beyond age 45 in either lineage”. This criterion could not reliably be assessed in this study population since pedigrees or other detailed description of family structure was not available for most patients. |
| - Triple negative breast cancer <=60y | 1st or 2nd degree relative with triple negative breast cancer <=60 |
| - Breast cancer and >=2 additional diagnoses of breast cancer at any age in patient and/or in close blood relatives*^b^* | 1st or 2nd degree relative with breast cancer and at least two additional diagnoses of breast cancer in that 1st or 2nd AND/OR in 1st 2nd or 3rd degree relative on the same side of the family*^d^* |
| - Breast cancer and >=1 close blood relatives*^b^* with breast cancer <=50y | 1st or 2nd degree relative with breast cancer and at least one additional 1st 2nd or 3rd degree relative on the same side of the family*^d^* with breast cancer <=50 |
| - Breast cancer and >=1 close blood relatives*^b^* with ovarian cancer | 1st or 2nd degree relative with breast cancer and at least one additional 1st 2nd or 3rd degree relative on the same side of the family*^d^* with ovarian cancer |
| - Breast cancer and a close male blood relative*^b^* with breast cancer | 1st or 2nd degree relative with breast cancer and at least one additional 1st 2nd or 3rd degree relative on the same side of the family*^d^* with male breast cancer |
| - Breast cancer and of Ashkenazi Jewish ancestry | NOT APPLIED - complete race/ethnicity data is not generally available for relatives. |
| - Ovarian cancer | 1st or 2nd degree relative with ovarian cancer |
| - Male breast cancer | 1st or 2nd degree relative with male BC |
| - Prostate cancer (Gleason score>=7) at any age with >=1 close blood relative*^b^* with ovarian cancer | 1st or 2nd degree relative with prostate (Gleason score>=7) and at least one additional 1st 2nd or 3rd on the same side of the family*^d^* with ovarian |
| - Prostate cancer (Gleason score>=7) at any age with >=1 close blood relatives*^b^* with breast cancer <=50y | 1st or 2nd degree relative with prostate (Gleason score>=7)and at least one additional 1st 2nd or 3rd on the same side of the family*^d^* with BC <=50 |
| - Prostate cancer (Gleason score>=7) at any age with >=2 close blood relatives*^b^* with breast cancer or prostate cancer (any grade)* at any age | 1st or 2nd degree relative with prostate (Gleason >=7) at any age with at least two additional 1st 2nd or 3rd on the same side of the family*^d^* with breast or prostate at any age |
| - Prostate cancer (Gleason score>=7) at any age with >=1 close blood relative*^b^* with pancreatic cancer | 1st or 2nd degree relative with prostate (Gleason >=7) at any age with at least one additional 1st 2nd or 3rd on the same side of the family*^d^* with pancreatic cancer |
| - Prostate cancer (Gleason score>=7) at any age with >=1 close blood relatives*^b^* with metastatic prostate cancer at any age | 1st or 2nd degree relative with prostate (Gleason >=7) at any age with at least one additional 1st 2nd or 3rd on the same side of the family*^d^* with metastatic prostate at any age |
| - Metastatic prostate cancer | 1st or 2nd degree relative with metastatic prostate |
| - Pancreatic cancer | 1st or 2nd degree relative with pancreatic cancer |
| - Prostate cancer (Gleason score>=7) at any age and Ashkenazi Jewish ancestry | NOT APPLIED - complete race/ethnicity data is not generally available for relatives. |
| - *BRCA1/2* mutation detected by tumor profiling in the absence of germline mutation analysis | NOT APPLIED - Similar to the scenario of a known familial mutation in the family, patients would likely undergo confirmatory testing for a single variant in this scenario unless otherwise indicated based on other clinical history factors. |
| *^a^*Adapted with permission from the NCCN Clinical Practice Guidelines in Oncology (NCCN Guidelines®) for Genetic/Familial High-Risk Assessment: Breast and Ovarian V.1.2019. © 2018 National Comprehensive Cancer Network, Inc. All rights reserved. The NCCN Guidelines® and illustrations herein may not be reproduced in any form for any purpose without the express written permission of NCCN. To view the most recent and complete version of the NCCN Guidelines, go online to NCCN.org. The NCCN Guidelines are a work in progress that may be refined as often as new significant data becomes available. | |
| *^b^*Close blood relative includes 1st, 2nd or 3rd degree relatives | |
| *^c^*Family history criteria were only evaluated for unaffected patients, where “unaffected” is defined as unaffected by any *BRCA1/2*-related cancers (breast, ovarian, prostate, or pancreatic cancer)  *^d^*Ordering clinicians were asked to specify maternal vs. paternal relationships on test requisition forms. In addition, clinical data was curated in a manner that designated maternal vs. paternal lineage. | |
