# Supplementary material for: A clinical guide to hereditary cancer panel testing: evaluation of gene-specific cancer associations and sensitivity of genetic testing criteria in a cohort of 165,000 high-risk patients
Source: Genet Med. 2019 Aug 13;22(2):407–15. doi: 10.1038/s41436-019-0633-8 (PMC7000322; doi:10.1038/s41436-019-0633-8)
Supplement: Supplementary file 3 — Supplementary Table S3 [file 41436_2019_633_MOESM3_ESM.docx]

Table S3. Application of NCCN genetic testing criteria for Lynch syndrome

| Criterion*^a^* | Description of exceptions and/or interpretations made in the application of criterion |
| --- | --- |
| Known lynch syndrome in the family | NOT APPLIED - In this scenario it is recommended to perform genetic testing for the familial mutation. Since the study population was patients who underwent multigene panel testing (and not testing for a single mutation), we did not evaluate this criterion. |
| Individual with colorectal cancer diagnosed <50y |  |
| Individual with endometrial cancer diagnosed <50y |  |
| Individual with colorectal cancer and another synchronous or metachronous LS-related cancer*^b^* |  |
| Individual with endometrial cancer and another synchronous or metachronous LS-related cancer*^b^* |  |
| Individual with colorectal cancer and >=1 first-degree or second-degree relative with LS-related cancer*^b^* diagnosed <50y |  |
| Individual with endometrial cancer and >=1 first-degree or second-degree relative with LS-related cancer*^b^* diagnosed <50y |  |
| Individual with colorectal cancer and >=2 first-degree or second-degree relatives with LS-related cancers*^b^* | Individual with colorectal cancer and >=2 first-degree or second-degree relatives on the same side of the family*^d^* with LS-related cancers*^b^* |
| Individual with endometrial cancer and >=2 first-degree or second-degree relatives with LS-related cancers*^b^* | Individual with endometrial cancer and >=2 first-degree or second-degree relatives on the same side of the family*^d^* with LS-related cancers*^b^* |
| Individual with colorectal cancer showing evidence of mismatch repair deficiency by MSI or IHC |  |
| Individual with endometrial cancer showing evidence of mismatch repair deficiency by MSI or IHC |  |
| Family history of >=1 first-degree relative with colorectal or endometrial cancer dx <50y |  |
| Family history of >=1 first-degree relative with colorectal or endometrial cancer and another synchronous or metachronous LS-related cancer*^b^* |  |
| Family history of >=2 first-degree or second-degree relatives with LS-related cancer*^b^*, including >=1 diagnosed <50y | Family history of >=2 first-degree or second-degree relatives on the same side of the family*^d^* with LS-related cancer*^b^*, including >=1 diagnosed <50y |
| Family history of >=3 first-degree or second-degree relatives with LS-related cancers*^b^* | Family history of >=3 first-degree or second-degree relatives on the same side of the family*^d^* with LS-related cancers*^b^* |
| Individual with an LS-related cancer*^b^* or unaffected individual with >=5%*^c^* risk of having an MMR gene mutation based on predictive models (PREMM5, MMRpro, MMRpredict) | Applied published PREMM5 algorithm*^e^* only |
| Individual with a colorectal tumor with MSI-high (MSI-H) histology (ie presence of tumor-infiltrating lymphocytes, Crohn's-like lymphocytic reaction, mucinous/signet ring differentiation, or medullary growth pattern) diagnosed <=60y | NOT APPLIED - While clinicians are prompted to provide results from MSI or IHC testing on tumors if available, we do not routinely request or receive pathology reports, therefore, we are unable to assess whether MSI-H histology is present. |
| *^a^*Adapted with permission from the NCCN Clinical Practice Guidelines in Oncology (NCCN Guidelines®) for Genetic/Familial High-Risk Assessment: Colorectal V.1.2018. © 2018 National Comprehensive Cancer Network, Inc. All rights reserved. The NCCN Guidelines® and illustrations herein may not be reproduced in any form for any purpose without the express written permission of NCCN. To view the most recent and complete version of the NCCN Guidelines, go online to NCCN.org. The NCCN Guidelines are a work in progress that may be refined as often as new significant data becomes available. | |
| *^b^*Lynch syndrome (LS)-related cancers include colorectal, endometrial, gastric, ovarian, pancreas, ureter and renal pelvis (kidney), brain, biliary tract, small intestine, sebaceous adenoma, sebaceous carcinoma, and keratoacanthoma  *^c^*There are recent data that resulted in a lower threshold of ≥2.5% for the PREMM5 predictive model risk for having an MMR gene mutation. Based on these data, it is reasonable for testing to be done based on the ≥2.5% score result and clinical judgment. Of note, with the lower threshold, there is an increase in sensitivity, but a decrease in specificity. It is not known how this applies to the general population of unaffected individuals.  *^d^*Ordering clinicians were asked to specify maternal vs. paternal relationships on test requisition forms. In addition, clinical data was curated in a manner that designated maternal vs. paternal lineage. | |
| *^e^*Kastrinos F, Uno H, Ukaegbu C, et al. Development and Validation of the PREMM5 Model for Comprehensive Risk Assessment of Lynch Syndrome. J Clin Oncol. 2017;35(19):2165-2172. | |
